# Isolating the delay component of impulsive choice in adolescent rats

**DOI:** 10.3389/fnint.2014.00003

**Published:** 2014-01-27

**Authors:** Jesse McClure, Jeffrey Podos, Heather N. Richardson

**Affiliations:** ^1^Neuroscience and Behavior Program, University of Massachusetts AmherstAmherst, MA, USA; ^2^Biology Department, University of Massachusetts AmherstAmherst, MA, USA; ^3^Psychology Department, University of Massachusetts AmherstAmherst, MA, USA

**Keywords:** impulsive choice, delay discounting, peak interval, adolescence, sex differences

## Abstract

Impulsive choice—the preference for small immediate rewards over larger delayed rewards—has been linked to various psychological conditions ranging from behavioral disorders to addiction. These links highlight the critical need to dissect the various components of this multifaceted behavioral trait. Delay discounting tasks allow researchers to study an important factor of this behavior: how the subjective value of a rewards changes over a delay period. However, existing methods of delay discounting include a confound of different reward sizes within the procedure. Here we present a new approach of using a single constant reward size to assess delay discounting. A complementary approach could hold delay constant and assess the utility of changing quantities of a reward. Isolating these behavioral components can advance our ability to explore the behavioral complexity of impulsive choice. We present in detail the methods for isolating delay, and further capitalize on this method by pairing it with a standard peak interval task to test whether individual variation in delay discounting can be explained by differences in perception of time in male and female adolescent rats. We find that rats that were more precise in discriminating time intervals were also less impulsive in their choice. Our data suggest that differences in timing and delay discounting are not causally related, but instead are more likely influenced by a common factor. Further, the mean-level change in our measure between post-natal day 28 and 42 suggests this test may be capturing a developmental change in this factor. In summary, this new method of isolating individual components of impulsive choice (delay or quantity) can be efficiently applied in either adolescent or adult animal models and may help elucidate the mechanisms underlying impulsivity and its links to psychological disorders.

## Introduction

Impulsive choice—the preference for small immediate rewards over larger delayed rewards—has been linked to substance abuse and addiction (Madden et al., 1997; Poulos et al., 1998; Mitchell, 1999; Mitchell et al., 2005; Field et al., 2007; Belin et al., 2008; Diergaarde et al., 2008; Oberlin and Grahame, 2009; Broos et al., 2012; Smith and Boettiger, 2012; Pattij and De Vries, 2013) and other psychological disorders such as attention deficit hyperactivity disorder (Winstanley et al., 2006) and schizophrenia (Heerey et al., 2007). These connections highlight the importance of exploring the mechanisms that govern impulsive choice, especially for adolescent populations (Whelan et al., 2012). However, measuring impulsive choice in adolescent animals can be challenging due to the brief duration within which animals can be pre-trained and tested for measures of impulsive choice. Another more general challenge is in interpretation of results from existing tests as there are two distinct causes of a preference for a small immediate reward over a larger delayed reward: either an increased aversion to delay, or a decreased sensitivity to reward size. These two potential drivers of impulsive choice are confounded in common behavioral tasks. We therefore present an approach that allows for the isolation and separate quantification of each component. The method for isolating delay is elaborated upon and applied below.

**Figure 1:**
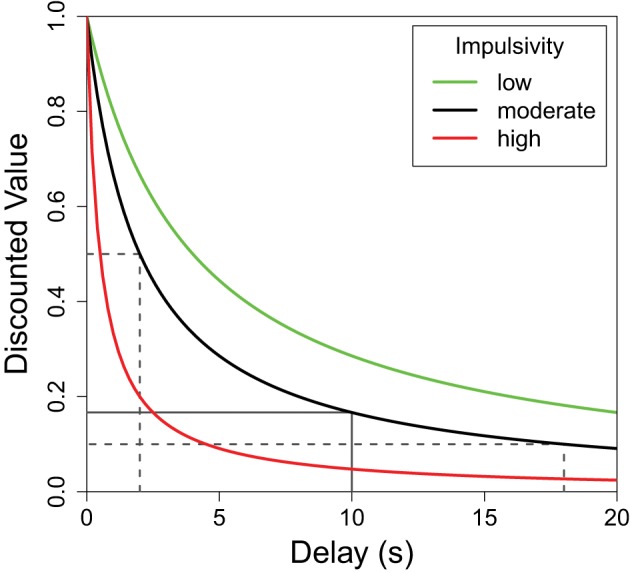
**The subjective value of a delayed reward**. The discounted value of a delayed reinforcer is modeled by a hyperbolic function. Here *f*(delay) = (1 + *K* × delay)^−1^ where “*K*” defines the shape of the discounting curve (green: *K* = 0.25, black: *K* = 0.5, red: *K* = 2.0). Higher *K*-values (red) are characteristic of greater impulsive choice. In each case, the average of the values of the variable options—shown, for the black function, in dotted lines—is greater than the value of their average which is shown as a solid line. This difference (Jensen's inequality) holds for any concave up function, but the difference is greater for functions with higher *K*-values. In other words, the difference between the variable and fixed delay options is greatest for the red line, moderate for the black line, and least for the green line.

Organisms generally prefer shorter delays and larger reward quantities, but the relationship between the perceived value of a reward and either of these parameters is not linear (Kahneman and Tversky, 1979; Bateson, 2002). More specifically, while the value of a reward continues to decrease as the delay increases, the greatest decrease in value is during the initial delay. This delay discounting relationship can be seen in Figure 1 where the greatest decrease in reward value occurs on the left side of the plot. For any non-linear function such as this, the average result of the function applied to two delay values differs from the result of the same function applied to the average of the two delays. To illustrate, the average discounted value of a delay alternating between 2 and 18 s (dotted lines in Figure 1) is greater than the discounted value of the same reward delayed 10 s (solid line). This mathematical property, known as Jensen's inequality (Jensen, 1906), is relevant to any study of variable properties and has been finding increasingly broad applicability within biology (Smallwood, 1996; Ruel and Ayres, 1999). Following the predictions of Jensen's inequality, organisms are often seen to prefer variable delays over fixed delays with the same average duration.

Although there is a general trend to prefer variable delays, the strength of this preference can differ greatly between individuals. Differences in the sharpness of the delay discounting curve (Figure 1) can be used to describe these individual differences. For illustrative purposes, Figure 1 shows the delay functions of three hypothetical subjects: red, black, and green. All three subjects would value a variable delay over the fixed delay, but the red subject presents a strong preference for the variable option (more impulsive), while the green subject presents a weaker preference for variability (less impulsive). As such, for each curve, a fixed delay less than 10 s would have the same discounted value as the average of the discounted values of 2 and 18 s, and this fixed delay would be lowest for the red curve, moderate for the black, and highest for the green curve.

This timing information, along with reward size, contributes to behavior in typical delay discounting tasks. Common behavioral assays of delay discounting provide variable and fixed delay options with different reward sizes for each (Ainslie, 1975; Dalley et al., 2011). As neither variable of delay or quantity is held constant, however, it cannot be known which is driving the resulting preference. While the mean adjusting delay task (Mazur, 1988) does hold the quantity constant across trials, it offers a choice between two options of differing reward quantity. Indeed, a recent study (Madden et al., 2011) questions whether preferences for variable delays correlate with impulsive choice. The failure to find such a correlation may be—as noted by the authors—due to preferences being influenced not only by delay, but also by reward size in the compound impulsive-choice task. These data further highlight the need to disentangle the delay and quantity parameters to further our understanding of impulsive choice, particularly in adolescent populations that show greater sensitivity to reward size (Laviola et al., 2003; Van Leijenhorst et al., 2010).

We therefore take an alternative approach inspired by parallel work in behavioral ecology (Caraco et al., 1980; Caraco, 1981; Real et al., 1982; Real and Caraco, 1986) that maintains a constant reward size—and uses the same reward for each choice option—and systematically reduces the delay on the fixed lever (now an adjusting “stable” lever) to the point at which it has the same subjective value as the variable option. This point of equal preference, or *equivalence point* (Mazur, 1984), allows precise determination of the shape of each subject's delay discounting curve while using the same reward quantity for all options. We also optimized pre-training procedures to allow the method to be practical for use with early adolescent rodent models.

We apply this method to test two hypotheses on the source of individual variation in delay discounting. First, delay discounting scores may be driven by differences in time perception. If so, a decreased ability to precisely discriminate time intervals should lead to lower delay discounting scores in our task. If an animal were unable to discriminate between any time intervals it would not detect a difference between the variable and fixed option and its choice would be random. Similarly, any imprecision in time perception can be expected to partially mask the expression of a preference for variable delays; animals that are less precise may thus show lesser delay discounting. Alternatively, greater timing ability and less extreme delay discounting could each be considered products of a healthy or well-developed nervous system and if found together might indicate a shared neurological mechanism for the two processes. Our findings suggest that differences in time perception do not drive differences in delay discounting. Instead, there is likely a common biological mechanism that enhances time perception and also increases the ability to wait longer for a reward in adolescent animals.

## Materials and methods

### Animals

Wistar rats (21 males, 19 females) were shipped with mothers from Charles River (Wilmington, MA) and arrived on post-natal day (PD) 18. Rats were kept on a 12–12 h light dark cycle (lights on from 8 am–8 pm). On PD 21 they were weaned and separated into same-sex cages of 3 juveniles per cage. Animals were fed *ad libitum* rat chow and had free access to food and water throughout all stages of training and testing. Animals acclimated to human contact by a minimum of 5 min of handling per day. All animal procedures were approved by the University of Massachusetts Amherst Institutional Animal Care and Use Committee.

### Operant pre-training

Prior to the onset of adolescence (PD23–PD27), rats were pre-trained to lever press in operant boxes in overnight sessions using a fixed ratio 1 (FR1) reinforcement schedule for 0.1 ml sweetened water (3% glucose/0.125% saccharin/tap water) as previously described (Gilpin et al., 2012). The same sweetened water reward was used for all following procedures. In the present study, pre-training sessions were conducted for 8 h overnight during the rats' dark/active period. A session was divided into 5-min bouts separated by a 1-min pauses in which the levers were retracted. Each bout was randomly assigned by the operant software to present the left lever, right lever, or both levers simultaneously. The single lever bouts ensured that rats sampled both levers. The session terminated when either 8 h had passed or the rat had received 300 rewards, whichever came first.

**Figure 2:**
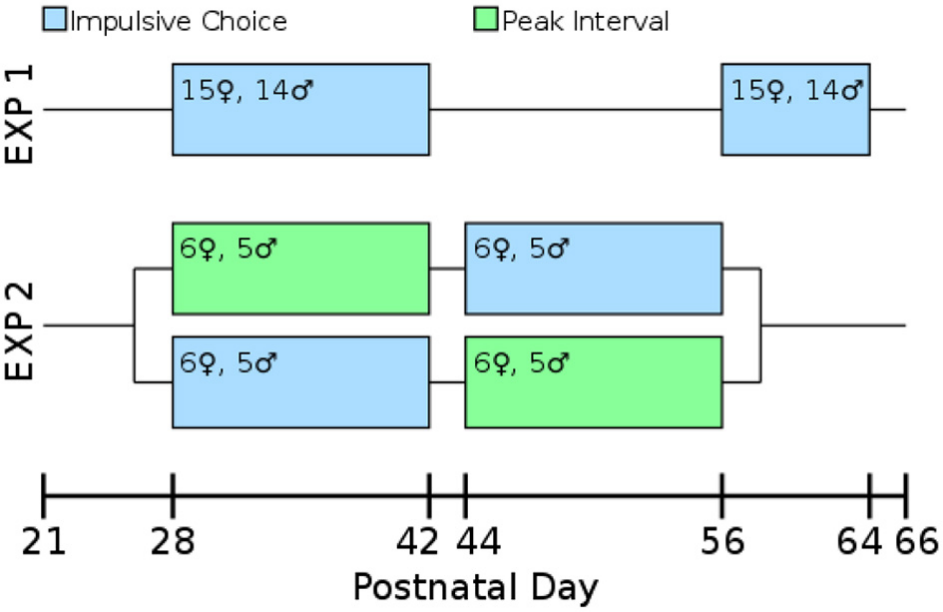
**Timeline and experimental design**. All measures were taken during adolescence or early adulthood. The test-retest consistency of the impulsive choice scores was assessed in Experiment 1: rats were tested from post-natal day (PD) 28–42 and retested from PD 58 to 64. The correlation between impulsive choice and peak interval performance was measured in a counterbalanced design: one cohort of the rats was tested for impulsive choice from PD 28 to 42 and peak interval performance from PD 44 to 56, the other cohort was the reverse.

Our laboratory has found that allowing multiple rats to run together in a single box for their first training sessions leads to improved performance in operant tasks. On PD 23 rats ran 3-per-box on the FR1 pre-training task. On PD 24–26 they were run 2-per-box switching partners each night. On PD 27 they ran singly which allowed assessment of their baseline lever pressing. By PD 27 all rats were actively pressing levers for the sweetened water reward. Impulsive choice (Experiments 1 and 2) or peak interval tasks (Experiment 2) began on PD 28. An overview of the design of both experiments is presented in Figure 2.

### Experiment 1—test-retest reliability

Experiment 1 evaluated the test-retest validity of our impulsive choice measure. High reliability should indicate the measure is assessing a trait or characteristic of the individual as opposed to being driven by their current state. Twenty-nine adolescent rats (14 males, 15 females) were tested for delay discounting as described below during early adolescence (PD 28–42) and again in early adulthood (PD 58–64).

#### Delay discounting testing

**Figure 3:**
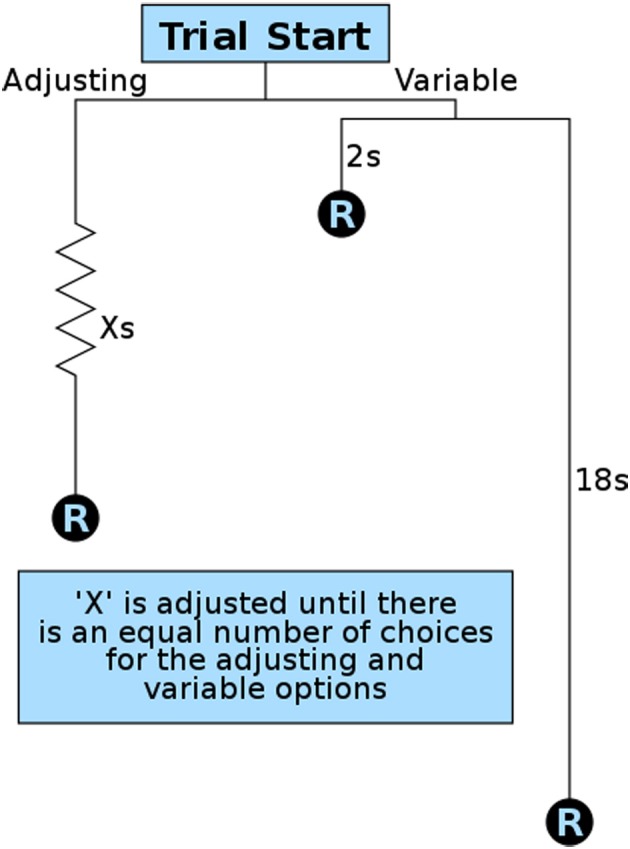
**Overview of a single impulsive choice trial**. Each free choice trial in the operant test presents the subject with a choice for a stable/adjusting delay reward (R) and a variable delay reward. The variable delay is set randomly per trial to 2 or 18 s. The adjusting delay (X) is adjusted after 21 free trials based on a subject's preference: 12 or more choices of the variable option result in a decrease in the stable delay; 9 or fewer choices of the variable option result in an increase in the stable delay. The amount the adjusting delay is reduced from the initial value of 10 s is used as an animal's delay discounting score.

To quantify delay discounting (a component of impulsive choice), we developed an operant method modeled after variance sensitive foraging (Stephens et al., 2007) and operant equivalence point tasks (Mazur, 1984, 1986; Bateson and Kacelnik, 1996, 1997). We designed our method for maximum efficiency so that animals could be studied during the brief window of adolescence, though the same procedures have also been used with naive adult animals. This important developmental period lasts approximately 4 weeks in rats and mice, with pubertal maturation occurring in the first half and brain development continuing on through the second half (Spear, 2000; Smith, 2003; Sisk and Zehr, 2005). We therefore ran this operant test for 8 h during rats' dark (active) period every night for 2 weeks. At the start of the first session the stable lever (left lever for half of the rats, right for the other half) was set to a 10-s delay. The session began with 9 forced trials in which only one lever (randomly selected per trial) was available to ensure the rats were sampling both levers. After having sampled both contingencies, rats were allowed 21 free trials in which their preferences could be expressed as elaborated below. Under these test conditions our animals quickly showed preferences for the variable lever, consistent with previous findings (Bateson and Kacelnik, 1995, 1998). Once this preference for the variable lever was expressed, the delay on the fixed (now “stable”) lever was gradually reduced over subsequent trials (Figure 3). This is in contrast to other methods that increase the quantity or concentration of the reward on the fixed lever. By decreasing the delay, the quality and quantity of all rewards remained constant throughout training and testing, and only delay was manipulated.

Training continued in repeating cycles of 9 forced trials and 21 free trials. The delay on the stable lever, set by the customized operant software, was reduced (−1 s) if the animal showed a preference for the variable lever (12 or more of the 21 presses on the variable lever) and increased (+1 s) if they showed a preference for the stable lever (9 or fewer presses on the variable lever). After each adjustment the subject was given another 9 forced trials so they could sample the new delay contingencies before another round of free trials. Training continued in 9 + 21 trial cycles throughout the night until either 8 h had passed or the rat received 300 rewards.

Every trial—forced sampling and choice trials—was initiated by the presentation of the lever and terminated when the rat pressed either lever. Upon a lever press, both levers were retracted and a stimulus light above the selected lever was illuminated throughout the pre-reward delay period. After the presentation of the reward, there was a 5 s intertrial interval. No post-reward delay, other than the fixed intertrial interval, was used.

On each subsequent night (8 pm) the delay on the stable lever was set to start where it ended the previous night. When a rat's stable delay lever remained constant due to the rat choosing either option equally often (or if it oscillated between two adjacent values), the adjustment step-size was reduced. At this point the operant software increased or decreased the stable lever by 0.5 s instead of 1 s in response to the rat's preference. Over subsequent nights this step-size was further reduced to increase the precision on the stable lever at which the rat had an equivalent preference for the variable and stable levers. The final delay on the stable lever was the rat's *equivalence point* (Mazur, 1986).

The degree to which the stable lever needed to be decreased (10 s minus the equivalence point) was used as our *delay discounting score*. This is effectively the same as the equivalence point, but has an advantage for interpretation given that a higher value indicates greater delay discounting (the more “impulsive” choice). This offset (“*x*” in Equations 1 and 2) score can be used to calculate an individual's rate-constant (*K*) for delay discounting.

As described earlier, delay discounting follows a hyperbolic decay (Figure 1) modeled by Equation 1 (Bateson and Kacelnik, 1998). The above method of testing impulsive choice is modeled by Equation 2: the delay on the stable lever is reduced by an offset (*x*) such that the animal assigns an equal value to this stable delay as to the average of the variable delay options. In the above described method the delay (*d*) is 10 s, and the variability (Δ) is 8 s. *K* is each individual's discounting rate constant; higher *K*-values represent more rapid delay discounting. An individual's *K*-value can be calculated from their offset (impulsive choice) score by Equation 3. The α parameter is a small constant, often set to 1 in many studies.

(1) $$\text{Value}=\frac{1}{\alpha +K\times d}$$

(2) $$\frac{2}{\alpha +K(d-x)}=\frac{1}{\alpha +K(d-\Delta )}+\frac{1}{\alpha +K(d+\Delta )}\;$$

(3) $$K=\frac{a\times x}{\Delta^{2}-d\times x}$$

#### Data analysis

Individual consistency, or test-retest validity, was assessed by regressing post-test scores of delay discounting (10 minus the equivalence point) on pre-test scores. The sex of the animal was included to assess for sex-specific differences or interaction effects. All analyses were conducted in the R statistical software package (R Core Team, 2012).

### Experiment 2—timing and delay discounting

Experiment 2 tested the prediction that lesser precision in timing would correlate with lower levels of delay discounting. Twenty-two rats (10 males, 12 females) were tested on delay discounting (following the same methods as Experiment 1) and peak interval performance (described below) during early (PD 28–42) and late (PD 42–56) adolescence (Spear, 2000; Smith, 2003; Sisk and Zehr, 2005). This experiment was counterbalanced by testing half of the rats on delay discounting first followed by peak interval, and the other half receiving the peak interval followed by our delay discounting task.

#### Peak interval testing

A peak interval task was used to assess accuracy and precision in discriminating time intervals. This task used a fixed interval (FI) 11 s reinforcement schedule in which the first response after 11 s is rewarded with the same sweetened water reward used in other procedures. There was no punishment nor response cost for early responses, and the response lever remained available throughout a trial. Under fixed interval schedules, organisms tend to begin responding at a low rate and increase response frequency as the criterion (11 s) approaches.

Peak interval was conducted in three stages each night in the same operant boxes used in other procedures but using only the right lever for all animals. In the first stage, a trial was initiated by the presentation of a single lever. After the criteria time (11 s) had elapsed, the stimulus light above the lever was illuminated indicating the availability of the reward. The second stage changed only in the absence of the stimulus light but maintained the same FI-11 criterion. The third stage was identical to the second, but included probe trials.

Probe trials were interspersed randomly with fixed interval trials. Probe trials were identical to fixed interval trials except that the reward mechanism was disabled. Response frequencies in probe trials tend to increase as the criterion time approaches, then taper off when no reward is delivered. Pooled across probe trials, response frequency distributions resemble a normal distribution. For a given subject, the average of this distribution indicates the accuracy with which they can discriminate the criterion interval, with the variance indicative of their precision.

Rats began peak interval testing on either PD 28 or PD 44, depending on whether they were tested before or after impulsive choice, respectively (Figure 3). Testing was conducted during the rats' active period (dark phase) every night for 7 days. Probe trials were interspersed with fixed interval trials by random selection (without replacement) for 1 out of 8 trials to be a probe trial. As the rats needed time to learn the task, data from the first two nights were not included in the analysis. Data on the time of each response—measured from the start of the trial—from the last five nights was pooled from all probe trials for each rat. The mean and standard deviation of all responses were used as measures of an animal's accuracy and precision in timing respectively.

#### Data analysis

Delay discounting scores (10 minus the equivalence point) were regressed on interval standard deviation (precision) scores from the peak interval task, to estimate the proportion of variance in delay discounting accounted for by differences in perceptual discrimination and test the prediction of lesser precision correlating with lower delay discounting. Sex was included as a control variable in all analyses to allow for the detection of sex-specific effects or interactions. All analyses were conducted in the R statistical software package (R Core Team, 2012).

## Results

### Experiment 1—test-retest reliability

**Figure 4:**
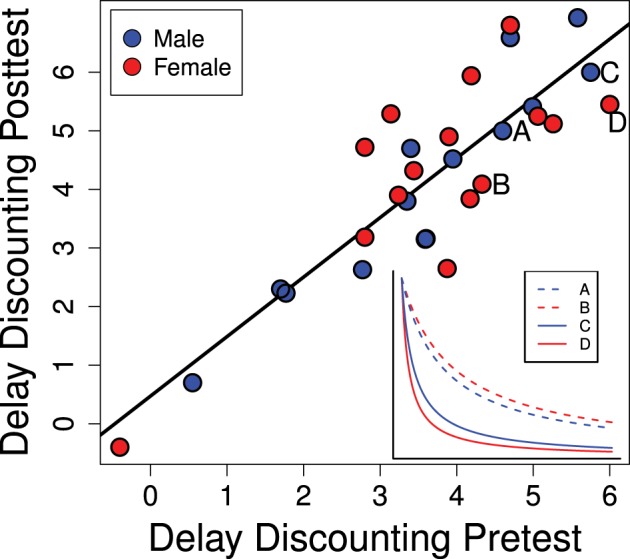
**Delay discounting scores in the pre- and post-tests (PD 28–42 and PD 58–64) are strongly correlated (*R*^2^ = 0.758; *p* < 0.0001)**. The inset graph shows representative delay discounting curves from two males and two females. Delay discounting scores are the degree to which the stable lever had to be reduced: higher scores indicate more rapid delay discounting which would be characteristic of a more impulsive choice.

Individual equivalence point scores in Experiment 1 were highly consistent over time as seen in Figure 4 (*R*^2^ = 0.758; *p* < 0.0001). This suggests that our method is detecting a repeatable trait of the individual. As this trait is the degree to which the value of a reinforcer declines with delay (delay discounting) it can be regarded as an index of impulsive choice. While this rank-order consistency was high, we also detected a mean-level increase in delay discounting scores between the pre- and post-tests [*t*_(28)_ = 3.29; *p* = 0.0027] suggesting a developmental or potentially experience-dependent increase in discounting rates. This change from pre- to post-test was similar for males and females [mean increase for males: 0.49 ± 0.18 and females: 0.57 ± 0.27; *t*_(27)_ = 0.25; *p* = 0.80].

### Experiment 2—timing and impulsive choice

**Figure 5:**
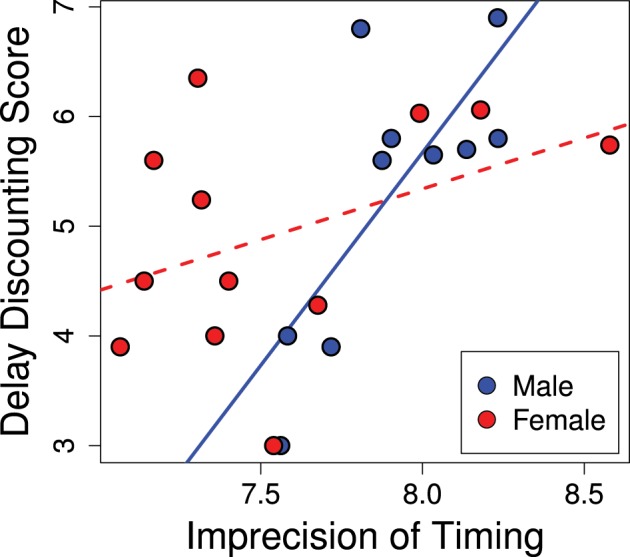
**Imprecision in timing predicts delay discounting**. Greater variance in the estimation of the interval in the peak interval task was positively correlated with greater discounting scores (*R*^2^ = 0.40; *p* = 0.02). This effect was driven by the males (males *b* = 3.88; *p* = 0.007), while the females showed a trend in the same direction (females *b* = 0.93; *p* = 0.15).

The variance of responses (standard deviation) correlated positively with delay discounting scores, as seen in Figure 5 (*R*^2^ = 0.40; *p* = 0.02). Animals that scored as less precise (more variance) in their temporal discrimination abilities also showed greater discounting (*b* = 1.40; *p* = 0.015). This effect was stronger in males and the significance of the overall effect was driven by males (males *b* = 3.88; *p* = 0.007) while females trended in the same direction (females *b* = 0.93; *p* = 0.15). Thus, greater precision in interval timing correlated with lower delay discounting.

No relationship was found between mean response times on probe trials and delay discounting scores (*R*^2^ = 0.03; *p* = 0.73). Thus, in our sample, we found no relationship between delay discounting and the accuracy of timing. Neither did the total number of responses on probe trials significantly correlate with delay discounting (*R*^2^ = 0.21; *p* = 0.22).

Following Bateson and Kacelnik (1995) we also analyzed the relationship between the final equivalence point and the latency to respond for each option. As our task is defined as terminating when an equal preference is displayed, we sampled latencies from early in training (days 2 and 3) when the animals should have had sufficient experiences with the contingencies, but had not yet approached an equivalence point, as well as late in training (days 13 and 14) when their preferences were stabilizing. Median response latencies to individual levers did not correlate with final equivalence points, nor did differential latencies between the levers (all *p* > 0.5). However, overall median latency to respond correlated positively with equivalence points (*R*^2^ = 0.25; *p* = 0.048).

## Discussion

The goal of the current study was to present a method that isolates the delay component of impulsive choice, and to apply this method to explore a behavioral mechanism that may underlie delay discounting. Results from Experiment 1 suggest that the measure of delay discounting we introduce here provides a robust and state-independent index of an individual trait. Additionally, as shown in Experiment 2, 40% of the observed variability in this trait can be accounted for by individual differences in time-interval discrimination ability. This correlation, however, is in the opposite direction from what would be predicted if differences in delay discounting were driven by differences in timing. Instead, delay discounting and timing are likely each influenced by a common mediating factor. By using the present approach we were able to keep reward size constant, thus narrowing down the likely mechanisms driving decision making in our animals.

While the current study assessed only the delay component of impulsive choice, a similar logic could be applied to study the effects of varying quantities or rewards while keeping delay constant. Together these methods can disentangle the timing and reward size components of this multifaceted behavior. By isolating the delay component in the current study, we found that rats that show greater delay discounting also show less precision in a peak interval timing task. This finding is in contrast to what might be expected given that all subjects prefer the variable delay option: any deficiency in timing would be expected to mask this preference and lead to a less impulsive score. Poor performance on the timing task and greater discounting in the same subjects are thus indicative of a separate shared mediating factor. While peak interval allowed a preliminary test of these alternative hypotheses, the current finding may be best expanded by further exploring the relationship using delay discrimination tasks in place of peak interval. In either case, employing the above elaborated delay discounting task will allow for proper isolation of the timing component of the choice task.

### Individual and developmental differences

Although our tests were conducted during an active period of neurological development in rats, the individual differences detected were highly consistent from the pre- to the post-test (rank-order consistency) in both males and females despite the wide range of between-individual variability in this measure. These levels of consistency are greater than those observed for human personality traits (Roberts and DelVecchio, 2000). There were, however, also small but significant mean-level changes in the population between the two tests. Rats discounted delayed rewards more steeply in the test in early adulthood (PD 58–64) than they did in early adolescence (PD 28–42). While these data seem to run counter to a general trend of decreasing delay discounting through development (Green et al., 1994; Steinberg et al., 2009), it may be that early adolescence is a time of greater discounting after which further development would lead again to lower discounting rates. We cannot yet, however, rule out experience as a driver of greater discounting scores in this task. Future studies could include additional testing periods in early, mid, and late adulthood to help address this.

Various circuits that contribute to delay discounting are actively developing over this period of adolescence. For example, individual differences in delay discounting have been attributed to variation in dopamine signaling (Forbes et al., 2007) using traditional methods of testing delay discounting. More specifically, genotypic variation in the dopamine transporter (DAT) gene correlates with individual differences in impulsive choice in human studies (Paloyelis et al., 2010), while experimental increases of DAT expression in animal studies drive greater impulsive choice (Adriani et al., 2009).

Dopaminergic activity in the ventral tagmental area (VTA) correlates with reward-size prediction error in fMRI BOLD signals in humans (D'Ardenne et al., 2008) and in neural recordings in animal models (Bayer and Glimcher, 2005; Tobler et al., 2005). The VTA relays this signal to the nucleus accumbens (NAcc) (Glimcher, 2011) where the higher levels of DAT activity are associated with greater delay discounting. This dopamine reuptake has been suggested to underlie decaying reward value curves—such as those in Figure 1—and thus individual differences in DAT in the NAcc could create differences in the resulting delay discounting curves. Many components of the dopamine system in addition to DAT correlate with impulsive choice in adolescent populations, but DAT and the dopamine D4 receptor show the most consistent associations (Nemoda et al., 2011). This dopaminergic circuitry is known to be actively developing through adolescence making this developmental stage a sensitive period for addiction (Chambers et al., 2003; Kuhn et al., 2010; Nemoda et al., 2011; Smith and Boettiger, 2012).

Several previous authors have proposed learning mechanisms that pair such a decaying reward signal with a reverse replay of recent events (Dragoi et al., 2003; Foster and Wilson, 2006; Kühn and Stamatescu, 2007; Pennartz et al., 2009). For example, *in vivo* hippocampal recordings in maze-running rats have shown a pattern of rapid reward-induced reverse-order replays of place cells (Foster and Wilson, 2006). These replays, or hippocampal ripples, have been further characterized and found to be coincident with similar ripple patterns in other brain regions including the NAcc (Goto and O'Donnell, 2001; Malhotra et al., 2012). As the hippocampal input to the NAcc can provide a temporal context (O'Donnell, 1999) it has been further suggested that this input can also provide more general contextual information (Goto and O'Donnell, 2001).

The correlation between decreased precision in timing and greater delay discounting that we observe suggests a correlated regulation of the dopamine signaling and timing circuitry that is commonly seen in schizophrenia (Heerey et al., 2007; Bonnot et al., 2011). The proposed circuitries for delay discounting and timing are also disrupted in animal models of schizophrenia (Kato et al., 2011; Nason et al., 2011). Schizophrenia in turn, has been proposed to be the result of abnormalities in the reorganization of this circuitry during adolescence (Feinberg, 1983; Jaaro-Peled et al., 2009). There may be variations in the development of this same circuitry in healthy populations as well; these variations may contribute to the correlation of timing and delay discounting we observe, and could also contribute to the mean-level change in delay discounting between our two testing periods.

In addition to the potential developmental effects, performance on the peak interval task is likely modulated by hormonal effects. Ovarian hormones are known to affect interval timing (Ross and Santi, 2000; Morofushi et al., 2001; Sandstrom, 2007; Pleil et al., 2011; Williams, 2012) such that intact females at different stages of their cycle may perform notably differently. This may account for the lack of a significant relationship between peak interval timing and our delay discounting score in females—as can be seen in Figure 5 where females show a wider range of imprecision in timing than males. This can be contrasted to delay discounting scores which have a comparable range in males and females (present study; Cross et al., 2011). Thus, we suspect the lack of a significant correlation in females is due to noise in the peak interval data rather than the delay discounting data.

### Challenges in interpreting compound choice tasks

The primary motivation in presenting the current method is to isolate the distinct components that contribute to impulsive choice. Impulsive choice is defined as a preference for a small immediate reward over a larger delayed reward; such a preference, however, can be the result of an aversion to delay, or an insensitivity to differing reward sizes. By isolating each factor, we hope to better characterize choice behavior in these tasks which may aid in understanding some conflicting results in the literature.

When presented with repeated choices for a small immediate reward or a larger delayed reward animals' responses may be influenced both by the delay discounting and quantity valuing utility curves. These choices, however, may also be affected by a number of other factors. Animals may assess the long-term rate of intake for each option which may bias them toward the small immediate reward as they could receive more of these in the allotted time. To account for this, most studies that present large delayed rewards and small immediate rewards add a post-reward delay to set a fixed trial duration regardless of which option is selected. However, recent findings have questioned the validity of this approach Blanchard et al. (2013).

As our method isolates a single parameter—either delay or quantity—no post-reward buffer was required. The present data in fact argue against long-term rate estimations driving animals' decisions in this study. At the start of testing, the expected long-term rate for either option would be equivalent. As all animals showed a preference for the variable option, the delay on the stable option was decreased. At this point the stable option would provide a *greater* long-term rate; yet the rats still favored the variable delay.

Hayden et al. (2008) argue that such a preference need not be due to a discounting curve, but may rather be indicative of a preference for uncertainty itself. By controlling for the predicted influence of utility curves these researchers demonstrated that discounting itself was insufficient to fully explain choice behavior while their subjects showed a strong preference for uncertainty. Our current data does not rule out such an interpretation. Whether the preference is due to the reinforcing properties of delayed rewards, or to the effect of variability itself, the present method allows for the quantification of this preference without any confound from the quantity of the reward.

In summary, many factors influence preference in the types of choice tasks described above. Different procedures and different testing conditions may isolate or accentuate some of these factors over others. Only by developing methodological tools to isolate each factor can researchers proceed to design rigorous empirical tests of the varied decision theories. We do not suggest that delay discounting is the sole, or even primary factor in such choices. The above presented method, however, will be an important tool for isolating the delay (or quantity) discounting components which will allow tests of the magnitude—or lack thereof—of their influence on decision making.

### Clinical applications

While humans and non-human animals show similar patterns in peak interval testing (Rakitin et al., 1998), it has been suggested that humans and animals differ in impulsive choice. Specifically, Kahneman and Tversky (1979) proposed prospect theory as a better explanatory model of inter-temporal choice in humans. While there is no doubt about this model's effectiveness, the differences from the models of animal behavior may have less to do with species differences as with differences in the test. Many human tasks designed to explore delay discounting use secondary reinforcers (e.g., money) and hypothetical scenarios. These conditions require a different set of cognitive processes than those required for the decision tasks employed in animal studies. When the hypothetical scenarios are replaced by primary reinforcers, human subjects' responses follow similar patterns as those of non-human primates (Hayden and Platt, 2009). Thus, these methods may be an effective tool in human studies provided the human subjects' task is framed appropriately. This could allow clinicians to explore mechanisms of addiction with these methods while the predictions of prospect theory may still serve best in modeling human choice in economic settings.

### Conclusions

The delay discounting method we introduce here provides advantages over current methods as subjects' responses to delays and quantities can be assessed independently. By separately assessing how variability in delay or variability in reward size contribute to the correlations observed in psychological disorders, researchers can focus on the neurological pathways that may be most relevant. Further, these methods have been optimized to be used in early adolescent rats. Such an approach will provide valuable insight into the mechanisms of addiction and may inform intervention and treatment methods.

### Conflict of interest statement

The authors declare that the research was conducted in the absence of any commercial or financial relationships that could be construed as a potential conflict of interest.

## References

[B1] Adriani W. Boyer F. Gioiosa L. Macri S. Dreyer J. L. Laviola G. (2009). Increased impulsive behavior and risk proneness following lentivirus-mediated dopamine transporter over-expression in rats' nucleus accumbens. Neuroscience 159, 47–58 10.1016/j.neuroscience.2008.11.042 19135135

[B2] Ainslie G. (1975). Specious reward: a behavioral theory of impulsiveness and impulse control. Psychol. Bull. 82, 463–496 10.1037/h0076860 1099599

[B3] Bateson M. (2002). Recent advances in our understanding of risk-sensitive foraging preferences. Proc. Nutr. Soc. 61, 509–516 10.1079/PNS2002181 12691180

[B4] Bateson M. Kacelnik A. (1995). Preferences for fixed and variable food sources: variability in amount and delay. J. Exp. Anal. Behav. 63, 313–329 10.1901/jeab.1995.63-313 7751835 PMC1334448

[B5] Bateson M. Kacelnik A. (1996). Rate currencies and the foraging starling: the fallacy of the averages revisited. Behav. Ecol. 7, 341–352 10.1093/beheco/7.3.341

[B6] Bateson M. Kacelnik A. (1997). Starlings' preferences for predictable and unpredictable delays to food. Anim. Behav. 53, 1129–1142 10.1006/anbe.1996.0388 9236010

[B7] Bateson M. Kacelnik A. (1998). Risk-sensitive foraging: decision making in variable environments, in Cognitive Ecology, ed Dukas R. (Chicago, IL: University of Chicago Press), 297–341

[B8] Bayer H. M. Glimcher P. W. (2005). Midbrain dopamine neurons encode a quantitative reward prediction error signal. Neuron 47, 129–141 10.1016/j.neuron.2005.05.020 15996553 PMC1564381

[B9] Belin D. Mar A. Dalley J. Robbins T. Everitt B. (2008). High impulsivity predicts the switch to compulsive cocaine-taking. Science 320, 1352–1355 10.1126/science.1158136 18535246 PMC2478705

[B10] Blanchard T. C. Pearson J. M. Hayden B. Y. (2013). Postreward delays and systematic biases in measures of animal temporal discounting. Proc. Natl. Acad. Sci. U.S.A. 110, 5491–15496 10.1073/pnas.1310446110 24003113 PMC3780845

[B11] Bonnot O. Montalembert M. d. Kermarrec S. Botbol M. Walter M. Coulon N. (2011). Are impairments of time perception in schizophrenia a neglected phenomenon? J. Physiol. Paris 105, 164–169 10.1016/j.jphysparis.2011.07.006 21803155

[B12] Broos N. Diergaarde L. Schoffelmeer A. Pattij T. De Vries T. (2012), Trait impulsive choice predicts resistance to extinction and propensity to relapse to cocaine seeking: a bidirectional investigation. Neuropsychopharmacology 37, 1377–1386 10.1038/npp.2011.323 22318198 PMC3327843

[B13] Caraco T. (1981), Energy budgets, risk and foraging preferences in dark-eyed juncos (junco hyemalis). Behav. Ecol. Sociobiol. 8, 213–217 10.1007/BF00299833

[B14] Caraco T. Martindale S. Whittam T. (1980). An empirical demonstration of risk-sensitive foraging preferences. Anim. Behav. 28, 820–830 10.1016/S0003-3472(80)80142-4

[B15] Chambers R. A. Taylor J. R. Potenza M. N. (2003). Developmental neurocircuitry of motivation in adolescence: a critical period of addiction vulnerability. Am. J. Psychiatry 160, 1041–1052 10.1176/appi.ajp.160.6.1041 12777258 PMC2919168

[B16] Cross C. P. Copping L. T. Campbell A. (2011), Sex differences in impulsivity: a meta-analysis. Psychol. Bull. 137, 97–130 10.1037/a0021591 21219058

[B17] D'Ardenne K. McClure S. M. Nystrom L. E. Cohen J. D. (2008). Bold responses reflecting dopaminergic signals in the human ventral tegmental area. Science 319, 1264–1267 10.1126/science.1150605 18309087

[B18] Dalley J. Everitt B. Robbins T. (2011). Impulsivity, compulsivity, and top-down cognitive control. Neuron 69, 680–694 10.1016/j.neuron.2011.01.020 21338879

[B19] Diergaarde L. Pattij T. Poortvliet I. Hogenboom F. de Vries W. Schoffelmeer A. (2008). Impulsive choice and impulsive action predict vulnerability to distinct stages of nicotine seeking in rats. Biol. Psychiatry 63, 301–308 10.1016/j.biopsych.2007.07.011 17884016

[B20] Dragoi V. Staddon J. E. Palmer R. G. Buhusi C. V. (2003). Interval timing as an emergent learning property. Psychol. Rev. 110, 126–144 10.1037/0033-295X.110.1.126 12529059

[B21] Feinberg I. (1983). Schizophrenia: caused by a fault in programmed synaptic elimination during adolescence? J. Psychiatr. Res. 17, 319–334 10.1016/0022-3956(82)90038-3 7187776

[B22] Field M. Christiansen P. Cole J. Goudie A. (2007). Delay discounting and the alcohol stroop in heavy drinking adolescents. Addiction 102, 579–586 10.1111/j.1360-0443.2007.01743.x 17309540

[B23] Forbes E. Brown S. Kimak M. Ferrell R. Manuck S. Hariri A. (2007). Genetic variation in components of dopamine neurotransmission impacts ventral striatal reactivity associated with impulsivity. Mol. Psychiatry 14, 60–70 10.1038/sj.mp.4002086 17893706 PMC2668513

[B24] Foster D. Wilson M. (2006). Reverse replay of behavioural sequences in hippocampal place cells during the awake state. Nature 440, 680–683 10.1038/nature04587 16474382

[B25] Gilpin N. Karanikas C. Richardson H. (2012). Adolescent binge drinking leads to changes in alcohol drinking, anxiety, and amygdalar corticotropin releasing factor cells in adulthood in male rats. PLoS ONE 7:e31466 10.1371/journal.pone.0031466 22347484 PMC3275622

[B26] Glimcher P. W. (2011). Understanding dopamine and reinforcement learning: the dopamine reward prediction error hypothesis. Proc. Natl. Acad. Sci. U.S.A. 108(Suppl. 3), 15647–15654 10.1073/pnas.1014269108 21389268 PMC3176615

[B27] Goto Y. O'Donnell P. (2001). Synchronous activity in the hippocampus and nucleus accumbens *in vivo*. J. Neurosci. 21, 1529–2401 11160416 10.1523/JNEUROSCI.21-04-j0003.2001 PMC6762233

[B28] Green L. Fry A. F. Myerson J. (1994). Discounting of delayed rewards: a life-span comparison. Psychol. Sci. 5, 33–36 10.1111/j.1467-9280.1994.tb00610.x

[B29] Hayden B. Platt M. (2009). Gambling for gatorade: risk-sensitive decision making for fluid rewards in humans. Anim. Cogn. 12, 201–207 10.1007/s10071-008-0186-8 18719953 PMC2683409

[B30] Hayden B. Y. Heilbronner S. R. Nair A. C. Platt M. L. (2008). Cognitive influences on risk-seeking by rhesus macaques. Judgm. Decis. Mak. 3, 389–395 19844596 PMC2763334

[B31] Heerey E. A. Robinson B. M. McMahon R. P. Gold J. M. (2007). Delay discounting in schizophrenia. Cogn. Neuropsychiatry 12, 213–221 10.1080/13546800601005900 17453902 PMC3746343

[B32] Jaaro-Peled H. Hayashi-Takagi A. Seshadri S. Kamiya A. Brandon N. J. Sawa A. (2009). Neurodevelopmental mechanisms of schizophrenia: understanding disturbed postnatal brain maturation through neuregulin-1–erbb4 and disc1. Trends Neurosci. 32, 485–495 10.1016/j.tins.2009.05.007 19712980 PMC2755075

[B33] Jensen J. (1906). Sur les fonctions convexes et les inégalités entre les valeurs moyennes. Acta Math. 30, 175–193 10.1007/BF02418571

[B34] Kahneman D. Tversky A. (1979). Prospect theory: an analysis of decision under risk. Econ. J. Econ. Soc. 263–291 10.2307/1914185

[B35] Kato T. Abe Y. Sotoyama H. Kakita A. Kominami R. Hirokawa S. (2011). Transient exposure of neonatal mice to neuregulin-1 results in hyperdopaminergic states in adulthood: implication in neurodevelopmental hypothesis for schizophrenia. Mol. Psychiatry 16, 307–320 10.1038/mp.2010.10 20142818

[B36] Kühn R. Stamatescu I. (2007). Learning with incomplete information and the mathematical structure behind it. Biol. Cybern. 97, 99–112 10.1007/s00422-007-0162-4 17534648

[B37] Kuhn C. Johnson M. Thomae A. Luo B. Simon S. A. Zhou G. (2010). The emergence of gonadal hormone influences on dopaminergic function during puberty. Horm. Behav. 58, 122–137 10.1016/j.yhbeh.2009.10.015 19900453 PMC2883625

[B38] Laviola G. Macrı S. Morley-Fletcher S. Adriani W. (2003). Risk-taking behavior in adolescent mice: psychobiological determinants and early epigenetic influence, *Neurosci.* Biobehav. Rev. 27, 19–31 10.1016/S0149-7634(03)00006-X 12732220

[B39] Madden G. J. Francisco M. T. Brewer A. T. Stein J. S. (2011). Delay discounting and gambling. Behav. Process. 87, 43–49 10.1016/j.beproc.2011.01.012 21352902 PMC3081402

[B40] Madden G. Petry N. Badger G. Bickel W. (1997). Impulsive and self-control choices in opioid-dependent patients and non-drug-using control patients: drug and monetary rewards. Exp. Clin. Psychopharmacol. 5, 256–262 10.1037/1064-1297.5.3.256 9260073

[B41] Malhotra S. Cross R. W. A. van der Meer M. A. A. (2012). Theta phase precession beyond the hippocampus. Rev. Neurosci. 23, 39–65 10.1515/revneuro-2011-0064 22718612

[B42] Mazur J. (1984). Tests of an equivalence rule for fixed and variable reinforcer delays. J. Exp. Psychol. Anim. Behav. Process. 10, 426–436 10.1037/0097-7403.10.4.426 3701262

[B43] Mazur J. (1986). Fixed and variable ratios and delays: further tests of an equivalence rule. J. Exp. Psychol. Anim. Behav. Process. 12, 116–124 10.1037/0097-7403.12.2.116 3701262

[B44] Mazur J. E. (1988). Esitmation of indifference points with an adjusting-delay procedure. J. Exp. Anal. Behav. 49, 37–47 10.1901/jeab.1988.49-37 3346621 PMC1338825

[B45] Mitchell J. Fields H. D'Esposito M. Boettiger C. (2005). Impulsive responding in alcoholics. Alcohol. Clin. Exp. Res. 29, 2158–2169 10.1097/01.alc.0000191755.63639.4a 16385186

[B46] Mitchell S. (1999). Measures of impulsivity in cigarette smokers and non-smokers. Psychopharmacology 146, 455–464 10.1007/PL00005491 10550496

[B47] Morofushi M. Shinohara K. Kimura F. (2001). Menstrual and circadian variations in time perception in healthy women and women with premenstrual syndrome. Neurosci. Res. 41, 339–344 10.1016/S0168-0102(01)00290-5 11755220

[B48] Nason M. W. Adhikari A. Bozinoski M. Gordon J. A. Role L. W. (2011). Disrupted activity in the hippocampal–accumbens circuit of type iii neuregulin 1 mutant mice. Neuropsychopharmacology 36, 488–496 10.1038/npp.2010.180 20927045 PMC3005939

[B49] Nemoda Z. Szekely A. Sasvari-Szekely M. (2011). Psychopathological aspects of dopaminergic gene polymorphisms in adolescence and young adulthood. Neurosci. Biobehav. Rev. 35, 1665–1686 10.1016/j.neubiorev.2011.04.002 21527290 PMC3133854

[B50] O'Donnell P. (1999). Ensemble coding in the nucleus accumbens. Psychobiology 27, 187–197 11797085

[B51] Oberlin B. G. Grahame N. J. (2009). High-alcohol preferring mice are more impulsive than low-alcohol preferring mice as measured in the delay discounting task. Alcohol. Clin. Exp. Res. 33, 1294–1303 10.1111/j.1530-0277.2009.00955.x 19389183 PMC2872785

[B52] Paloyelis Y. Asherson P. Mehta M. A. Faraone S. V. Kuntsi J. (2010). Dat1 and comt effects on delay discounting and trait impulsivity in male adolescents with attention deficit/hyperactivity disorder and healthy controls. Neuropsychopharmacology 35, 2414–2426 10.1038/npp.2010.124 20736997 PMC2955909

[B53] Pattij T. De Vries T. (2013). The role of impulsivity in relapse vulnerability, Curr. Opin. Neurobiol. 3, 700–705 10.1016/j.conb.2013.01.023 23462336

[B54] Pennartz C. Berke J. Graybiel A. Ito R. Lansink C. Van Der Meer M. (2009). Corticostriatal interactions during learning, memory processing, and decision making. J. Neurosci. 29, 12831–12838 10.1523/JNEUROSCI.3177-09.2009 19828796 PMC3849625

[B55] Pleil K. E. Cordes S. Meck W. H. Williams C. L. (2011). Rapid and acute effects of estrogen on time perception in male and female rats. Front. Integr. Neurosci. 5:63 10.3389/fnint.2011.00063 22016725 PMC3192991

[B56] Poulos C. Parker J. Le^ D. (1998). Increased impulsivity after injected alcohol predicts later alcohol consumption in rats: evidence for “loss-of-control drinking” and marked individual differences. Behav. Neurosci. 112, 1247–1257 10.1037/0735-7044.112.5.1247 9829802

[B57] Rakitin B. C. Gibbon J. Penney T. B. Malapani C. Hinton S. C. Meck W. H. (1998). Scalar expectancy theory and peak-interval timing in humans. J. Exp. Psychol. Anim. Behav. Process. 24, 15–33 10.1037/0097-7403.24.1.15 9438963

[B58] R Core Team (2012). R: a Language and Environment for Statistical Computing. Vienna: R foundation for statistical computing ISBN 3-900051-07-0.

[B59] Real L. Caraco T. (1986). Risk and foraging in stochastic environments. Annu. Rev. Ecol. Syst. 17, 371–390 10.1146/annurev.es.17.110186.002103

[B60] Real L. Ott J. Silverfine E. (1982). On the tradeoff between the mean and the variance in foraging: effect of spatial distribution and color preference. Ecology 1617–1623 10.2307/1940101

[B61] Roberts B. DelVecchio W. (2000). The rank-order consistency of personality traits from childhood to old age: a quantitative review of longitudinal studies. Psychol. Bull. 126, 3–25 10.1037/0033-2909.126.1.3 10668348

[B62] Ross L. Santi A. (2000). The effects of estrogen on temporal and numerical processing in ovariectomized female rats. Psychobiology 28, 394–405 10.3758/BF03331997

[B63] Ruel J. Ayres M. (1999). Jensen's inequality predicts effects of environmental variation. Trends Ecol. Evol. 14, 361–366 10.1016/S0169-5347(99)01664-X 10441312

[B64] Sandstrom N. J. (2007). Estradiol modulation of the speed of an internal clock. Behav. Neurosci. 121, 422–432 10.1037/0735-7044.121.2.422 17469932

[B65] Sisk C. Zehr J. (2005). Pubertal hormones organize the adolescent brain and behavior. Front. Neuroendocrinol. 26, 163–174 10.1016/j.yfrne.2005.10.003 16309736

[B66] Smallwood P. D. (1996). An introduction to risk sensitivity: the use of jensen's inequality to clarify evolutionary arguments of adaptation and constraint. Am. Zool. 36, 392–401

[B67] Smith C. T. Boettiger C. A. (2012). Age modulates the effect of comt genotype on delay discounting behavior. Psychopharmacology 222, 609–617 10.1007/s00213-012-2653-9 22349272 PMC3401276

[B68] Smith R. (2003). Animal models of periadolescent substance abuse. Neurotoxicol. Teratol. 25, 291–301 10.1016/S0892-0362(02)00349-5 12757826

[B69] Spear L. (2000). Modeling adolescent development and alcohol use in animals. Alcohol Res. Health 24, 115–123 11199278 PMC6713014

[B70] Steinberg L. Graham S. OBrien L. Woolard J. Cauffman E. Banich M. (2009). Age differences in future orientation and delay discounting. Child Dev. 80, 28–44 10.1111/j.1467-8624.2008.01244.x 19236391

[B71] Stephens D. Brown J. Ydenberg R. (2007). Foraging: Behavior and Ecology. Chicago, IL: University of Chicago Press 10.7208/chicago/9780226772653.001.0001

[B72] Tobler P. N. Fiorillo C. D. Schultz W. (2005). Adaptive coding of reward value by dopamine neurons. Science 307, 1642–1645 10.1126/science.1105370 15761155

[B73] Van Leijenhorst L. Zanolie K. Van Meel C. S. Westenberg P. M. Rombouts S. A. Crone E. A. (2010). What motivates the adolescent? brain regions mediating reward sensitivity across adolescence. Cereb. Cortex 20, 61–69 10.1093/cercor/bhp078 19406906

[B74] Whelan R. Conrod P. J. Poline J.-B. Lourdusamy A. Banaschewski T. Barker G. J. (2012). Adolescent impulsivity phenotypes characterized by distinct brain networks. Nat. Neurosci. 15, 920–925 10.1038/nn.3092 22544311

[B75] Williams C. L. (2012). Sex differences in counting and timing. Front. Integr. Neurosci. 5:88 10.3389/fnint.2011.00088 22319476 PMC3251826

[B76] Winstanley C. Eagle D. Robbins T. (2006). Behavioral models of impulsivity in relation to adhd: translation between clinical and preclinical studies. Clin. Psychol. Rev. 26, 379–395 10.1016/j.cpr.2006.01.001 16504359 PMC1892795

